# Experience of Filipinos with Spinal Cord Injury in the Use of Assistive Technology: An Occupational Justice Perspective

**DOI:** 10.1155/2020/6696296

**Published:** 2020-11-25

**Authors:** Daryl Patrick G. Yao, Kaoru Inoue, Michael P. Sy, Peter Bontje, Natsuka Suyama, Chiyomi Yatsu, Dante A. Perez, Yuko Ito

**Affiliations:** ^1^Department of Occupational Therapy, Graduate School of Human Health Sciences, Tokyo Metropolitan University, Tokyo, Japan; ^2^National Teacher Training Centre for the Health Professions, University of the Philippines, Manila, Philippines; ^3^Occupational Therapy Section, Department of Rehabilitation Medicine, Philippine Orthopaedic Centre, Quezon City, Philippines

## Abstract

Assistive technology (AT) is often required to facilitate the performance of occupations and promote inclusion and reduction of dependency among persons with spinal cord injury (SCI). However, only 5-15% of individuals in developing countries have access to AT. This study is aimed at exploring the experience of Filipinos with SCI as they use AT and understand these from an occupational justice (OJ) perspective. This study utilised a hermeneutic phenomenological approach to explore the participants' experiences with AT usage. Ten participants were recruited from a hospital and communities within Metro Manila, Philippines, and interviewed last January 2020. Hermeneutic analysis was done to interpret the shared meaning embedded within their experiences and was informed by an occupational justice perspective. Exploring the experience of the participants in using AT yielded four themes, namely, (1) engaging in occupations despite limited opportunities, (2) going to various locations amidst an inaccessible environment, (3) striving towards inclusion in spite of attitudinal barriers, and (4) securing needs in light of unfavourable life conditions. Filipinos with SCI deal with numerous structural and contextual factors in daily life. There has been partial enablement of OJ as they incorporate AT in their daily lives as occupational rights are far from being recognised and respected. In infusing an OJ perspective to understanding AT use, OT practitioners are bound to identify problems and courses of action that go beyond traditional service delivery.

## 1. Introduction

Persons with spinal cord injury (SCI) face multiple hindrances in daily life such as limited mobility and assistive product equipment, environmental and contextual challenges, and financial issues [1–8]. These hindrances subject them to a lower rate of economic participation, significantly higher healthcare costs, and societal exclusion [9, 10]. These situations are more pronounced in developing countries, such as the Philippines, wherein persons with SCI experience a significantly lower state of well-being and reduced access to work, education, health, social welfare, and disaster management compared to individuals without disability [2, 11, 12].

Assistive technology (AT) is a “broad range of devices, services, strategies, and practices that are conceived and applied to ameliorate problems” ([13], p. 460). It is often required to facilitate the performance of occupations and reduction of dependency among persons with SCI [10]. However, only 5-15% of individuals with disabilities in developing countries, such as the Philippines, have access to AT [10]. Among Filipinos who use a wheelchair as an AT device, only 58% uses the device for at least an hour daily and only 33% of total users can use their wheelchairs outdoors unassisted [14]. Hence, the importance of adequate service delivery to an increased frequency of wheelchair use indoors and outdoors and higher performance of daily activities was stressed [14, 15].

A health professional involved in the AT service delivery is occupational therapy (OT) practitioner. OT is a health profession that possesses knowledge and skills in client assessment and selecting the most appropriate AT [16]. An OT practitioners' competence in determining the person-environment-occupation interplay is necessary for effective AT interventions [17]. By identifying the impact of disability and barriers, OT practitioners could effectively render AT service provision to promote justice for their clients [17, 18].

AT is arguably a justice issue as it facilitates the equity of one's capability to pursue and achieve well-being [19]. The Convention on the Rights of Persons with Disabilities (CRPD), as well as various world organisations, asserted that AT is integral to supporting a person with disability (PWD) to a life without discrimination [20, 21]. A concept which resonates with the CRPD is occupational justice (OJ). OJ refers to the equity in rights of every individual to meet basic needs and to have equal and diverse opportunities to meet one's potential through participating in meaningful occupations and experience well-being [22]. According to the framework for OJ (FOJ), violations of OJ occur when structural and contextual factors hinder participation in occupation and the exercise of occupational rights [23]. The use of an OJ perspective in AT service delivery for SCI is a plausible approach to facilitate an equitable provision of AT, efficacious use of AT, and attainment of human rights.

While the literature revealed that AT consumers in America experience occupational injustices in AT service provision and use [24], there is a dearth of literature which discusses social and human right issues of AT users in developing countries [2]. Furthermore, OJ has not been explored in the Philippine setting outside the field of substance addiction rehabilitation [25]. While the National Unified Health Research Agenda has identified biomedical products, social determinants, and equity and health as research priorities [26], there were no opportunities for the OT profession to articulate neither AT usage nor the use of an OJ perspective for persons with SCI.

This study sought to answer the question “What is the experience of Filipinos with SCI in their use of AT?.” Exploring the experiences of Filipinos with SCI will provide OT practitioners' baseline information for determining strategies to support their clients with SCI, advocating their clients' equity and rights, promoting self-advocacy in asserting the right to choose and engage in diverse occupations that could fulfil their basic needs and future aspirations, and developing ideas and questions that can inform future research. This article is aimed at exploring the experiences of Filipinos with SCI as they use AT and understand these from an OJ perspective.

## 2. Method

### 2.1. Design

A phenomenological approach guided by the principles of van Manen [27] was utilised to explore and interpret the participants' experience of a certain phenomenon. van Manen [27] explained that every description entails an interpretation to describe a unique moment of experience. Following the methodological processes proposed by van Manen [27, 28], this study (1) turned towards the phenomenon related to AT use among Filipinos with SCI, (2) investigated experiences as lived by the participants, (3) reflected on the essential themes which characterise AT use as informed by an OJ perspective, (4) described the phenomenon through writing and rewriting, (5) maintained a strong and OJ-oriented relation to the phenomenon, and (6) balanced the research context by considering parts and whole.

### 2.2. Participants and Setting

Purposive sampling was done wherein the author attempted to recruit 11 participants. Among which, one individual declined without providing a reason. None of the participants dropped out of the study. Recruitment was done with the help of a government hospital (Philippine Orthopaedic Centre) and a nongovernment organisation (Life Haven Independent Living Centre) from the Philippines. The former is a government hospital in the Philippines that is tasked to provide services relating to musculoskeletal disorders and related condition, including SCI [29], while the latter is a movement made by persons with disability to assert their rights [30] and is the only nongovernment organisation to respond to the researcher's correspondence. Inclusion criteria were adults (1) with SCI, (2) aged 21 to 59 years old [31], and (3) use AT device/s that ameliorates problems caused by SCI to participate in occupations within one's natural context. Exclusion criteria were (1) individuals with other comorbid conditions that impair the ability to recollect and report experiences, (2) have difficulty in speech and communication, and (3) are medically unstable (suffering from a systemic infection or in critical condition) during data collection days. These factors were identified using the clinical eye of the primary investigator and local collaborator (licensed OT practitioners) and a culture-specific strategy of *pakikiramdam* (shared inner perception). Pakikiramdam is an essential ability in interacting with Filipinos who are used to an indirect, nonverbal manner of communicating with others [32].

### 2.3. Ethics

Ethics review and approval was secured from the Ethics Review Committee of Tokyo Metropolitan University (Acceptance number: 19070) and the Philippine Orthopaedic Centre. Participants were asked to sign an informed consent (IC) prior to the interview. In the case of inability to hold a pen for signature, a thumb mark from the participant was taken. The IC form provided author information, research information, and provisions (right to prematurely terminate the interview and the right to decline from answering questions they may perceive as sensitive).

### 2.4. Data Collection

From the 13^th^ to the 24^th^ of January 2020, three participants in the hospital were interviewed in their preferred environment (bedside or separate room), while seven community-dwelling participants were interviewed in their own homes or office during a predetermined schedule. Demographics collected include age, sex, civil status, province, socioeconomic status (household income per month), severity and level of SCI, years living with SCI, educational history, previous/current work, and system usability scale (SUS) score of AT use. The SUS is a short and easy ten-item, five-point Likert scale with a correlation to one aspect of usability—performance [33]. While the SUS is not yet established as a valid tool in the Philippines, it has been deemed as reliable and valid by numerous studies and is considered as an industry standard [34]. Acquiring this score can provide a glimpse of how the user perceives the usability of their AT device and aid practitioners to determining whether an AT device is acceptable [35].

After demographic collection, in-depth interview guided by the concepts and principles of human rights and OJ was conducted. The opening question was “Can you tell me how your everyday life went ever since you started using your <AT>.” Follow-up questions included are as follows: “how did it affect your attainment of human rights” and “can you say that you experience equal opportunity to engage in activities.” Probing questions were asked as needed to expound on some experiences. Face-to-face interviews lasted for 30 to 60 minutes. All interviews were audio-recorded and subsequently transcribed. “Taglish,” the Filipino language with a mixture of English, was used as it is an effective communicative strategy among Filipinos of the current century [36]. Interviews were done by the primary author.

Member checking, the method of returning an interview data to a participant [37], was done from the 27^th^ of January to the 7^th^ of February 2020. Transcripts were given to the participants to retract, expound, or alter any of their statements. Follow-up questions were then asked during this time. Follow-up questions were individualised per participant based on the first interview and were predetermined by the primary author and a local collaborator. Examples included “were there other devices or resources that were instrumental in your daily life” and “since you mentioned <issue>, can you expound on this based on your experience”. Four participants had face-to-face follow-up interviews while the rest of the participants were contacted through phone call in their preferred time to uphold participants' safety as cases of COVID-19 are being reported in the Philippines.

### 2.5. Analysis and Interpretation

Prior to analysis, transcribed interviews were translated into English, a shared language among the authors, to enable equal access and comprehension of the topic. The translation was based on the intended meaning of the interviewee rather than a literal translation. This is because the Filipino language uses unique words that serve as a substitute to words or ideas that are mutually known by people conversing by virtue of the context of its use [38]. The primary author translated, while another reviewed the translation, and both are Filipinos bilingual in both languages.

The analysis was based on van Manen's hermeneutic analysis. Data analysis includes the following: (1) uncovering thematic aspects, (2) isolating thematic statements, (3) composing linguistic transformations, and (4) gleaning thematic descriptions [28]. The phenomenon was described as themes to capture "experiential structures that make up that experience" ([28], p. 59). In the first phase, the English transcripts were read to uncover thematic aspects based on the whole. In the second phase, ATLAS.ti version 8.0 [39] was used to do open coding, which assisted in isolating thematic statements to identify codes representative of the phenomenon. During the third phase, themes were drafted based on the information from the first two phases. Lastly, a description which captures the essence of the whole and the parts was written.

### 2.6. Trustworthiness and Rigor

Lincoln and Guba [40] identified credibility, transferability, dependability, and confirmability as components of trustworthiness. This study employed various strategies outlined by Korstjens and Moser [41] to maintain trustworthiness. Member checking and triangulation of investigators during data analysis were done to uphold credibility. A thick description of the participants' characteristics was provided to enhance transferability judgment among readers. To uphold dependability and confirmability, an inquiry audit was made by an individual uninvolved in this project; audit trails were kept by the primary author, which included interview transcripts, translations, and interpretation; reflexivity was observed by maintaining a note that tackled the primary author's subjective response to the environment and prior relationship with participants.

It is important to disclose that three participants have been acquainted with the primary researcher in the fulfilment of his job 1-2 years prior to this project. One of which was a direct client of the primary researcher.

## 3. Findings

### 3.1. Participant Characteristics

Ten individuals with SCI participated in this study. Participants' age ranged from 24 to 57 years (*m* = 44). Majority of the participants were from the lower socioeconomic status residing in the urban area of Metro Manila. Eight of the participants were wheelchair dependent while two individuals could walk for short distances only. Nine participants identified a wheelchair as the AT device they possess and use, while one participant identified a walker. Results of the SUS and participant characteristics are summarised in Table 1.

The SUS score provided a glimpse of how they perceived the usefulness, and consequently, the appropriateness of an AT device. Their comments supported the validity of the scores; for instance, Aljur (31/M) who provided the lowest SUS score among the participants articulated, “the armrest and footrest are fixed so, I cannot use a transfer board. I am dependent on others for pressure relief. I am slouched because the wheelchair is not fit for me. Hence, I don't use the wheelchair often.” Individuals who acquired their AT through the government's social welfare services had the lowest SUS among participants. Additionally, everyday technology (cell phone) and social support (personal assistant and helpers) were equally important in maximising one's potential in the Philippines.

### 3.2. Filipinos with SCI's Experience in the Use of Their AT

Participants reported the use of mobility devices as a representation of AT. Four themes from the data collected revealed the experience of participants as they used AT to ameliorate the pressing challenges in their daily lives. These are the following: (1) engaging in occupations despite limited opportunities, (2) going to various locations amidst an inaccessible environment, (3) striving towards inclusion in spite of attitudinal barriers, and (4) securing needs in light of unfavourable life conditions. Based on their experiences, all participants are exposed to OJ and injustices in daily life as they use AT.

#### 3.2.1. Engaging in Occupations despite Limited Opportunities

The first theme elucidates how AT use may influence participants' opportunity to engage in occupations (i.e., ADL, IADL, work, leisure, and social participation). Opportunities to engage in several occupations (i.e., work and education), however, remain limited despite the use of appropriate and sufficient AT as the environment, organisation, and norm.

Participants recognised that having an appropriate AT is vital to engage in occupations. Ted (57/M) articulated that “with the use of the wheelchair, I can attend to community meetings, churches, family gatherings, reunions, and any invitations.” Other participants also included practicing one's profession (Jessica 56/F), maintaining the security of one's home (Jorge 48/M), shopping (Jeffrey 48/M and John 31/M), playing with pets (Jorge 48/M), engaging in religious activities (Tado 43/M), and enjoying the mall (Derek 51/M) as occupations experienced because of AT use.

AT use enables occupations that enhance health experiences. Tado (43/M) shared, “I can do things independently. With the electric wheelchair, I feel confident… I am not limited to the house.” The increase of confidence has a positive impact on his mental health as it changed his outlook in life by knowing he can still accomplish something. Additionally, the AT is a means to engage in health maintaining activities such as rehabilitative programs (Derek 51/M and Tim 24/M).

Regardless of having an appropriate AT device, opportunities for several occupations remain limited. Jorge (48/M), a medical doctor by profession, shared that

I was not able to formally return to studying [my second master's degree] because, when I inquired how the setup will be, they had reservations. While it is good that they considered certain angles by thinking ‘if you attend class on a wheelchair, you might face difficulties in attending classes' or ‘the teacher might have trouble adjusting to your needs', it reflected that the university and its people are not ready. The system is not familiar with how to accommodate PWDs to the learning environment.He attributed this issue as a concern that goes beyond AT use with its roots stemming from the Philippines' customs and culture. He said, “In the Philippines, it is rare to see a PWD to pursue education. Looking at society, you study and pursue skills training, and then, there is no next level. No workplace would hire you.” Limited opportunities to return to their previous work were a challenge the participants experienced (Jeffrey 48/M, John 31/M, and Jorge 48/M). Hence, some participants were forced to stop their job pursuits or were forced to explore new means to make a living.

#### 3.2.2. Going to Various Locations Amidst an Inaccessible Environment

The second theme describes how AT allows the participants to go to various places within the community. However, they expressed the existence of multiple challenges in terms of environmental accessibility including the following: public transportation, substandard roads and sidewalks, absence or unsafe ramps and lifts, and nonconforming physical layouts. These challenges limit their choice with regard to where they can go and what they can do.

Wheelchair, the AT device mostly used by the participants, is instrumental for their mobility. Aljur (31/M) shared that because of AT, “I can go out but near the vicinity.” This ability to go out within their vicinity allowed them to access establishments within the community such as the convenience store (John 31/M), wet market (Jeffrey 48/M), restaurants (Jorge 48/M), church (Tado 43/M), local government office (Jeffrey 48/M, Jorge 48/M), and the basketball court (Derek 51/M).

However, going out may put their lives at risk because the participants mentioned that they had to use the streets and highways due to poorly maintained sidewalks. Jeffrey (48/M) experienced an accident on the road and shared this incident, “I experienced being a victim of a hit-and-run accident. I was crossing the street. When I was in the middle of the road, the stoplight suddenly turned green. A car hit me and broke my wheelchair.” Other participants also voiced out their concern over their safety as they had to share the road with numerous vehicles (Ted 47/M and Zaldy 52/M) whenever they use their AT device to go somewhere.

In long-distance travels, prevailing public utility vehicles are not accessible even with AT use. Jessica (56/F) recalled an instance wherein she had to ride a jeepney, “It was difficult for me. My husband had to carry me to board and alight. Riding it was painful on the back, so my husband supported me.” Inaccessible transportation made the participants think twice before engaging in an activity as Aljur (30/M) shared, “There was a time I was invited to an art exhibit, but I couldn't go because of the transportation.”

Upon reaching their destination, several public buildings and spaces also pose a challenge for the participants. Ted (57/M) shared, “ramps do not have the right measurements. It is too steep, dangerous. There was a time wherein my personal assistant had to remove his footwear to prevent sliding as he pushed me up the ramp.” Participants also reported the absence of ramps in other buildings; hence, they would have to be carried up and down the stairs (Jorge 48/M and Zaldy 52/M). Apart from accessing the building, physical barriers also exist within the structures like what Jorge (48/M) experienced:

The space is limited so; it is difficult to aptly manoeuvre the wheelchair... So, if you are a wheelchair user, you can reach the restaurant, but the indoor space is not fine... You must check beforehand and inquire before going to a place.

#### 3.2.3. Striving towards Inclusion in spite of Attitudinal Barriers

In the third theme, AT device enabled participants to tackle issues concerning their assertion of individuality, empowerment of other PWDs, and alteration of the public's attitudes through being more visible and vocal in articulating their thoughts in the community. Facing stigma and negative perceptions have been part of their daily struggles. The society partly installs barriers which lead to a loss of identity and meaning derived from activities.

One way to experience social inclusion is through enjoying the rights and freedom granted by the country to its citizens. Zaldy (52/M) shared that AT has been vital to conduct an assembly and freely communicate their thoughts to others, including the government. An instance was when he, along with other wheelchair users, “assembled at a certain meeting place and rode our wheelchair towards the compound of the Commission of Human Rights to demand for accessibility.” The participants also identified asserting their right to vote (Derek 51/M and Zaldy 52/M) by going to the precinct as an experience they were able to do due to having an AT.

By having and using AT devices to promote inclusion, they were able to inspire and empower other PWDs as a by-product. Jeffrey (48/M) recollected, “wheelchair users who were ashamed to go out, went out. Because they saw me making a living on the streets, it gave them hope.” Furthermore, several participants (Jeffrey 48/M, Jessica 56/F, Jorge 48/M, and Zaldy 52/M) were able to serve as a mediator between the government and other people because of the visibility and voice they gained.

By utilising AT devices, the participants made others aware of their situation and altered their attitude. Zaldy (52/M) shared,

When I went out of the house to socialise with people around me, they learned how to interact with me. They had a lot of questions. I responded to it so that they can be aware of my circumstances. No matter the situation, you can still educate others. Impart to them what they need to know. While there are positive experiences with strangers (Aljur 31/M and Jorge 48/M), the participants unanimously mentioned how the negative attitude of others has a greater impact and makes full inclusion challenging. Participants experienced not being allowed to enter an establishment (John 31/M), asked to leave the business premises (Jorge 48/M), perceived as a beggar (Jeffrey 48/M), and belittlement (Derek 51/M) as struggles under this theme. In using his wheelchair to attend a meeting, Jorge (48/M) shared the following experience:

When I was waiting in the corridor, I was asked to leave. I replied, ‘Why are you asking me to leave? I have a meeting.' The mentality of the staff was that all people going there are there to beg, ‘You are just here to ask for assistance'… The staff insisted that I am blocking the corridor, a nuisance.

#### 3.2.4. Securing Needs in Light of Unfavourable Life Conditions

The final theme elucidates how participants utilise AT to acquire financial resources necessary in securing their needs in terms of health, shelter, and sustainable resources. While AT use is important in engaging in income-generating activities to augment their financial capacity, they need to endure unfavourable conditions such as the lack of work security and a lower amount of salary. Disability benefits and charitable handouts are necessary adjuncts to accomplish roles as a parent, employee, life partner, and member of the community.

AT has enabled a handful of participants to pursue work as a dentist, vendor, entrepreneur, and civil servant. Tado (43/M) remarked how having his electric wheelchair enabled him to “work and earn as normal.” Despite this, he still faces challenges as he disclosed:

My peers earn more than me because they can move well. I am not a regular worker. So, mine is just like an allowance, below the minimum wage. I also do not get any nightshift differential. However, if I do not have this sort of income, my wife and I cannot sustain our family… I must make sacrifices. I cannot buy the things my children need/want because I need to spend for my medical needs.Another challenge experienced by participants is the unsteady influx of income (Aljur 30/M and Jeffrey 48/M) which could have serious repercussions such as the inability to rent their home and sustain daily living. This necessitates the participants to rely on financial support from others. For instance, Tado (43/M) admitted to relying on his friend for the maintenance of his AT. He shared, “I have a friend who helps me buy the things needed for maintenance, like the battery. I am dependent on donations. I have no other sources.” In addition to receiving assistance from friends and charitable individuals (Aljur 30/M, John 31/M, and Jorge 48/M), participants also shared that they had to appeal and rely on government (Tado 43/M) and nongovernment entities such as politicians (Jeffrey 48/M), organisations (Tado 43/M), and religious groups (Jeffrey 48/M) to provide support in sustaining their needs.

## 4. Discussion

This study sought to explore the experience of Filipinos with SCI as they used AT in their daily lives. It can be surmised that as the participants utilise AT in their daily life, they only experienced partial enablement of OJ as their occupational rights are far from being recognised and respected. This section is written based on how the structural and contextual factors observed within the themes facilitated and hindered OJ in accord with the constructs of FOJ [23].

The study revealed that AT use is perceived as an enabler of occupation, a catalyst to acquire needs, and a capacitator in achieving inclusion. It is an important contextual factor which modifies one's experience on how structural factors contribute to OJ. For some occupations that are not heavily affected by structural factors (occupations within the home and immediate community), the themes revealed that the use of appropriate AT can capacitate individuals to influence occupational outcomes through the access of available resources. This is the role of AT argued by literature [3, 42]. AT use also enhances mobility as it allows the participants to go to various locations. As mobility is a key to occupations [7], we argue that AT is necessary to one's mobility, access, and choice. However, in the Philippines, the use of appropriate AT devices alone remains insufficient in ensuring positive occupational outcomes as other factors also play a vital role in shaping one's experience.

Besides AT, there are other factors facilitative of OJ. A structural factor found in this study is the Filipino traits of interdependence and helpfulness. Filipinos with SCI consider their social support (caregivers and personal assistants) as a necessary element in enabling OJ. Because attainment of total independence is not a priority within the non-Western world [43], receiving social support is not perceived as negative but a welcome adjunct to AT use in enabling engagement in occupation and towards inclusion. Additionally, charitable acts are well-received by the participants. Contrary to how Tanudtanud-Xavier [44] deemed the charitable mindset in society as a barrier to accessing basic services, this study found that receiving the charity of others is a necessary means to acquire AT, maintain AT, and diminish the unfavourable life conditions they face in their daily life.

Occupational injustices, on the other hand, were concurrently present in every theme. Structural factors contributing to the limited opportunities and attitudinal barriers experienced within the themes of engaging in occupations and striving for inclusion are the Philippines' customs and norms. Because of the well-accepted belief that PWDs are not expected to be financially and physically independent from their family [45], creating opportunities to engage in education and work are not prioritised. This study found that Filipinos with SCI perceive that the environment and administration are ill-prepared to accommodate them in the school or workforce. This finding justifies as to why most wheelchair users in the Philippines are either self-employed or gained employment in sheltered workshops [46]. Because AT use does not provide much opportunity for mainstream work and education, Filipinos with SCI may be forced to seek and apply to sheltered workshops which offer work unrelated to their previous job and expertise. Moreover, customs and norms are a viable cause as to why there is a lack of awareness among the public regarding disability issues in all sectors of society [44, 47]. As a result of seeing PWDs as someone to be sheltered with, there is a negative stereotype within the Philippines that PWDs have a low level of productivity and practice frequent absenteeism [48]. This may be a reason why Filipinos with SCI often struggle with attitudinal barriers as found in this study, supporting the attitudinal issues found to exist by various researchers [1, 5, 6, 12, 42].

A structural factor evident within the themes of going to various locations and securing needs that partially contributes to the inaccessible environment and unfavourable living conditions is the current policies. While current legislation [49, 50] clarified the rights of PWDs, the implementation of disability-related legislation has much to be desired [44, 51, 52]. Thus, the environment and mass transportation remain inaccessible to Filipinos with SCI, starkly contrasting the situations of their counterparts in developed countries wherein they can go to various places with the use of an electric scooter [42]. While there are accessible areas in the Philippines, most infrastructures add accessibility features haphazardly for the sake of minimum compliance [53]. Filipinos with SCI are also subjected to unsafe conditions, such as traffic accidents, thereby violating their right to safety and security. Additionally, disability-related benefits were perceived to be insufficient. While PWD in the Philippines enjoys discounts in availing basic commodities [52], employment support remains scarce, thereby impairing the capacity of Filipinos with SCI to engage in income-generating activities. The challenges in engaging in income-generating activities may have repercussions in one's daily life as nonprimary healthcare needs and daily commodities in the Philippines would necessitate a predominantly out-of-pocket expenditure [54]. Hence, this study bolsters the argument that the social inequalities existing in current living conditions affect the attainment of justice and human rights [55, 56].

### 4.1. Implications to Practice in the Philippines

OJ concepts can influence outcomes relative to social justice, such as human rights, as both concepts are interweaved [18, 57]. Thus, in exploring and understanding the experiences of Filipinos with SCI, we can identify gaps and problems stemming from the often-overlooked issues within the society such as policies and customs. It also revealed facilitators that can be tapped to maximise one's opportunity to experience OJ. Hence, adapting AT service delivery to the local context and infusing it with OJ concepts and framework can shed light on the often-overlooked structural and contextual factors.

Considering the experiences of the participants, OT practitioners play a vital role in AT service delivery and to bridge the Filipinos with SCI towards achieving OJ. Based on the findings of this study, OT practitioners should aid in equipping sources of charitable support with the proper knowledge and resources necessary for providing appropriate AT device that can empower Filipinos with SCI. Advocating for the adequate implementation of disability-related policies is recommended as these are necessary for enabling engagement of occupation and OJ. Additionally, Filipino OT practitioners should engage in collaborative practice to determine action plans and policies responsive to the plight of the population of concern. Collaboration has long been identified as a means towards social justice [58] and in securing OJ [57]. OT practitioners can engage in participatory approaches with Filipinos with SCI and other health and nonhealth professionals to promote social inclusiveness and accessibility. It is necessary for the disability sector, health sector, nonhealth sector, and policymakers to cocreate solutions to address the problems in AT service delivery for Filipinos with SCI and the attainment of OJ. Lastly, OT practitioners have a role to play in breaking existing preconceptions and stereotypes through educating the public regarding disability awareness. As the Philippines is the social media hub of the world [59], advocacy efforts and education could be done through the strategic use of mainstream and social media. Informing the public of the challenges and struggle of Filipinos with SCI, as well as the role of AT to capacitate them, can provide opportunities to understand and subsequently open opportunities towards a more inclusive society.

### 4.2. Limitations

Study limitations include social desirability bias, as three participants have been acquainted with the primary researcher in the fulfilment of his job years prior to this project. Furthermore, as several individuals are key figures in the organisation partnered with, their ideologies tend to resonate and agree well with one another. More heterogeneous participants might further elucidate other attributes related to the themes. Transferability is also limited to Filipinos with SCI who share similar characteristics to the participants of this study. Nevertheless, this study offers a systematic manner of understanding the phenomenon of people with SCI in the context of AT and offers a novel perspective in viewing their experiences through identifying elements of OJ and injustices.

As this has been the first study to frame SCI and AT from an OJ perspective in the Philippines, it is proposed that future research can (1) explore the generalisability of the experiences observed in this study, (2) develop a systematised manner of acquiring OJ-related data, and (3) investigate the feasibility of integrating an established AT evaluation and procurement system to the national health insurance.

## 5. Conclusion

AT is considered as an instrument to ameliorate the challenges of PWDs, including those with SCI, through facilitating one's capability to achieve OJ and participate in meaningful occupations. This study explored the experiences of Filipinos with SCI as they used AT. It was found that Filipinos with SCI can experience doing occupation, access several places, becoming a part of society, and securing their individual needs despite the existence of multiple challenges which limits total and optimal engagement. There has been partial enablement of OJ as they use AT in their daily lives as occupational rights are far from being recognised and respected. In using an OJ perspective, OT practitioners are bound to identify problems and courses of action that go beyond traditional service delivery.

## Figures and Tables

**Table 1:** Demographic information.

Pseudonyms	Age/sex/civil status	Sci level; AIS^∗^	Province	Work	Educational attainment	SES^∗∗^	AT device (years of experience)	Source	SUS score^∗∗∗^	Other AT & supports^^^
Aljur	30/M/cohabitating	C4; AIS-C	Metro Manila (urban)	Online seller	High school (not completed)	Low	Standard WC -No removable/adjustable parts (5 years)	Social welfare; donation (friend)	2.5, unacceptable	Caregiver (wife); mobile phone
Derek	51/M/married	C3; AIS-D	Metro Manila (urban)	Unemployed	Elementary	Low	Standard WC -No removable/adjustable parts (1.5 years)	Social welfare	60, marginal	Caregiver (wife)
Jeffrey	48/M/cohabitating	T12; AIS-A	Metro Manila (urban)	Vendor	Bachelors (not completed)	Low	Sports WC (25 years)	Donation (politician, church)	72.5, acceptable	Companion (wife) (for long-distance travels)
Jessica	36/F/married	T12; AIS-A	Metro Manila (urban)	Dentist	Doctor of dental medicine	Middle	Standard WC: removable armrest, movable footrest (26 years)	Health insurance (overseas)	95, acceptable	Personal assistant
John	31/M/married	C6; AIS-A	Cagayan Valley (rural)	Unemployed	High school	Poor	Standard WC, with air cushion *-*Removable armrest -Movable footrest (8 years)	Donation (friend)	52.5, marginal	Caregiver (wife)
Jorge	48/M/married	C4; AIS-B	Metro Manila (urban)	NGO worker	Doctor of medicine	Middle	Electric WC --&-- Reclining WC (20 years)	Out-of-pocket; donation (colleague)	82.5, acceptable	Personal assistant; mobile phone
Tado	43/M/married	T11; AIS-A	Metro Manila (urban)	Brgy. worker (radio operator)	Bachelors	Low	Electric WC --&-- Electric scooter (10 years)	Donation (relative)	100, acceptable	Caregiver (son) (for transfers only)
Ted	57/M/single	C5; AIS-B	Metro Manila (urban)	NGO worker	Bachelors (not completed)	Low	Reclining WC (20 years)	Out-of-pocket	85, acceptable	Personal assistant
Tim	24/M/single	C5; AIS-D	Metro Manila (urban)	Student	Bachelors (not completed)	Middle	Walker --&-- Standard WC -No removable/adjustable parts (2 years)	Accident compensation (private)	80, acceptable	Caregiver (maid); mobile phone
Zaldy	52/M/married	C5; AIS-B	Metro Manila (urban)	NGO worker	Bachelors (not completed)	Low	Reclining WC (16 years)	Out-of-pocket	72.5, acceptable	Personal assistant

^∗^American Spinal Injury Association Impairment Scale. ^∗∗^Socioeconomic status: poor: < PHP 9,520; low income: PHP 9,520-19,040; middle income: PHP 19,040-114,240; upper income: PHP 114,240-190,400 (Albert et al., 2018). ^∗∗∗^Acceptability measure: acceptable: above 70; marginal: 50-70; unacceptable: below 50 [35]. ^^^While all participants owned a mobile phone, only those who explicitly articulated its utility as it enables participation and rights were included in this table.

## Data Availability

Access to raw data is restricted due to legal and ethical concerns, particularly third-party rights and privacy. All other information data is embedded within the manuscript.
